# Ganglion Impar Block: A Magic Bullet to Fix Idiopathic Coccygodynia

**DOI:** 10.7759/cureus.33911

**Published:** 2023-01-18

**Authors:** Bhanu P Swain, Sri Vidhya, Sharad Kumar

**Affiliations:** 1 Anesthesiology, Tata Main Hospital, Jamshedpur, IND; 2 Anesthesiology, Manipal - Tata Medical College, Jamshedpur, IND

**Keywords:** idiopathic coccygodynia, coccydynia, local infiltration analgesia, ganglion impar block, trans-sacrococcygeal joint approach, intra-coccygeal joint approach

## Abstract

Coccygodynia (coccydynia) is a painful condition of the perineum in the region of the tailbone or coccyx, aggravated by sitting on hard surfaces. It is frequently associated with injuries to the coccyx following direct trauma. Nevertheless, idiopathic coccygodynia without antecedent trauma history is not uncommon. Most of these patients respond to anti-inflammatory medications and physical therapy. Those who are unresponsive may require additional intervention for pain relief. Blockade of ganglion impar, the terminal end of the pelvic sympathetic chain, can dramatically alleviate the pain in patients suffering from coccygodynia. In the current case series, four patients in the age range of 21 to 69 years suffering from chronic idiopathic coccygodynia (female: male ratio of 1:1) were treated with ganglion impar block. All four patients received a course of medical management, and two of the patients additionally received local infiltration of the coccyx before ganglion impar block administration. The block was performed with fluoroscopy guidance by either the trans-sacrococcygeal joint approach or the intra-coccygeal joint approach. The pre-intervention average numeric rating pain score (NRS) was 7.5. After a single ganglion impar block intervention, all four patients experienced complete pain relief (NRS=0). No patients required a repeat injection, and all were pain-free for the entire one-year follow-up period.

## Introduction

Coccygodynia (coccydynia) is a painful condition of the perineum first described by Simpson in the 19th century [1]. It is characterized by pain localized to the tailbone (coccyx), aggravated by sitting on hard surfaces [2]. Coccygodynia accounts for approximately 1%-3% of low back pain, though the exact incidence is unknown [3]. Women are more susceptible than men because of their typical perineal anatomy [4]. Obese populations are also commonly affected [5]. The average age of involvement is 40 years, though coccygodynia can occur in any age group [5].

There are multiple etiologies described in the literature. One of the consistent findings is a subluxated or hypermobile coccyx. Injuries of the coccyx are often caused by backward falls onto hard ground. Trauma during childbirth is another common antecedent factor. Idiopathic causes are also reported without a prior history of trauma. Rarely, malignant pathologies of the perineum cause coccygodynia [6].

The management of coccygodynia always begins with conservative therapies such as anti-inflammatory medications, physical therapy, mobilization of the coccyx, and stretching exercises [5]. Patients unresponsive to these therapies ultimately need invasive interventions. Several minimally invasive injection techniques are employed, such as local infiltration anesthesia of the coccyx, coccygeal nerve block, caudal epidural injection, and ganglion impar block [7]. Coccygectomy is done in a few selected cases. Given the high complication rate, surgery is considered only when other pain-relieving options are exhausted [5].

The ganglion impar block is the most promising of the minimally invasive injection techniques [8]. Ganglion impar (Walther Ganglion) is the terminal sympathetic ganglion. It is located in the retroperitoneal space, posterior to the rectum and anterior to the coccyx. Ganglion impar block can be easily performed fluoroscopically, though computer tomography (CT) imaging and ultrasound guidance can also be used. There are multiple fluoroscope-guided techniques described in the literature. The one most commonly followed is the trans-sacrococcygeal technique.

Neurolytic ganglion impar block is usually reserved for coccygodynia of malignant etiology [9]. Radiofrequency ablation (RFA) of ganglion impar can also be performed. It provides long-standing pain relief in chronic coccygodynia [10]. The usual recommendation is to treat cases of recurrent pain after the ganglion impar block with local anesthetics and steroids. This case series highlights the effect of ganglion impar block on chronic idiopathic coccygodynia.

## Case presentation

Four patients (two males and two females) between the ages of 21 and 69 presented to our pain clinic with pain around the tailbone (Table 1). The duration of the pain varies from four months to one year. There was no history of direct trauma to the coccyx in any of the patients. Local perineum examination revealed a normal coccyx in all four cases except the fourth, where the coccyx was hypermobile. A radiograph of the pelvis with lateral projection was done in all cases to rule out a fracture.

**Table 1:** Patient characteristics *diabetes mellitus; **low back pain; *** hypertension

Case	Age	Sex	BMI	Comorbidity	Pathogenesis	Duration of pain
1	55	F	30.2	DM*, LBP**	Idiopathic	6 Months
2	21	M	22.6	-	Idiopathic	4 Months
3	61	M	29.4	DM	Idiopathic	1 year
4	69	F	24	DM, HTN***, LBP	Idiopathic	4 Months

Initially, all the patients were put on non-steroidal anti-inflammatory drugs (NSAIDS) (piroxicam 20 mg once daily for ten days), sitz bath treatment, use of a doughnut cushion while sitting on hard surfaces, and perineal stretching exercises for at least two weeks. None of the patients significantly responded to conservative therapy and later received local coccygeal infiltration, ganglion impar block, or both. Physical visits or phone calls were used to follow up on the patients one year after the intervention. All the patients underwent routine investigations before intervention. Informed consent was obtained for the procedure from all patients before any intervention. The interventions were daycare procedures, and the patients went home the same day after one hour of observation. The first and second cases initially received local infiltration of the coccyx and later underwent ganglion impar block. The third and fourth cases exclusively received ganglion impar block. Local infiltration anesthesia provided good short-term pain relief in the first and second cases, but the pain recurred. However, a single shot of ganglion impar block produced long-term pain relief (NRS= 0) until one year of follow-up (Table 2).

**Table 2:** Treatment modalities and pain scores at different periods *local coccygeal Infiltration; **ganglion impar block; ***numeric rating score

Case	Intervention	Pre-procedure pain (NRS***)	Post-procedure pain in NRS				
			First week	First month	Third month	Sixth month	One year
1	Local infiltration*	8	2	4	6	7	-
	GIB** (after six months)	7	0	0	0	0	0
2	Local infiltration*	7	0	6	7	-	-
	GIB (after four months)	7	0	0	0	0	0
3	GIB	7	0	0	0	0	0
4	GIB	9	0	0	0	0	0

**Figure 1:**
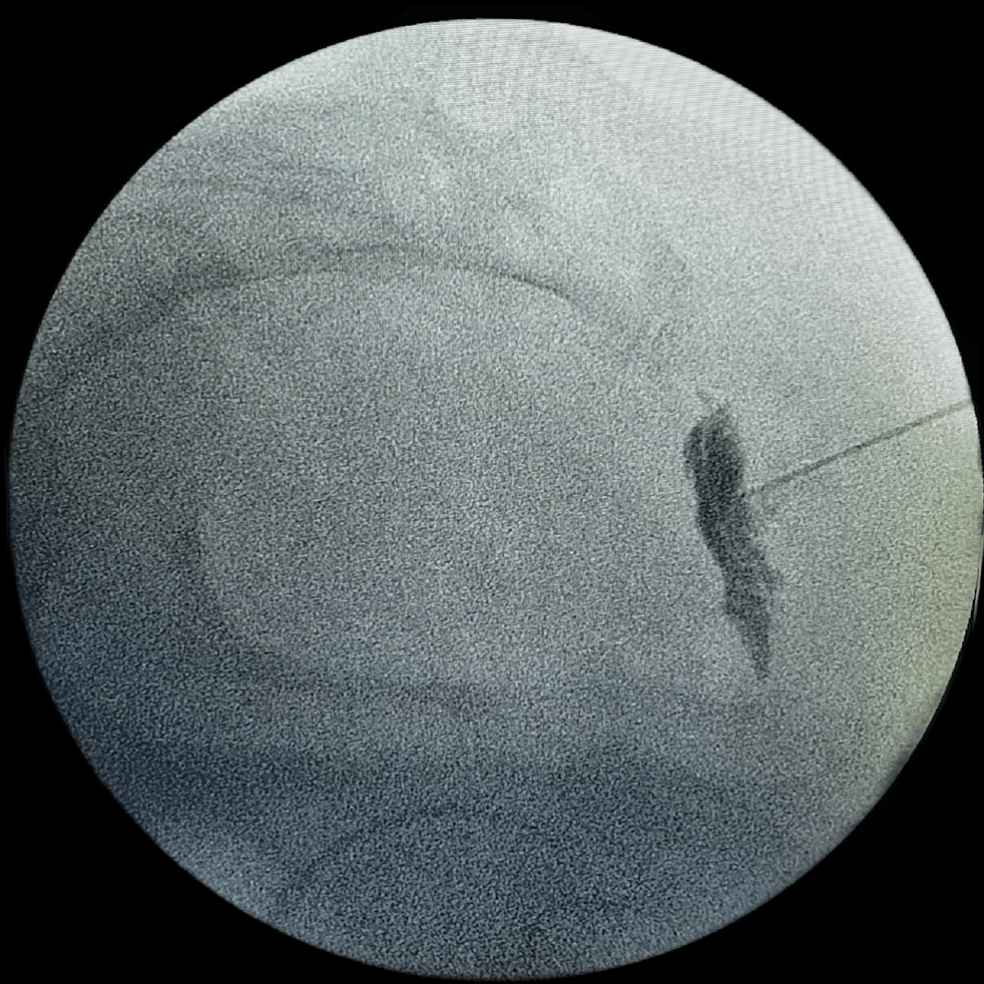
Lateral view of the fluoroscopic image showing needle insertion at the sacrococcygeal joint and the injected contrast appearing like an inverted comma

**Figure 2:**
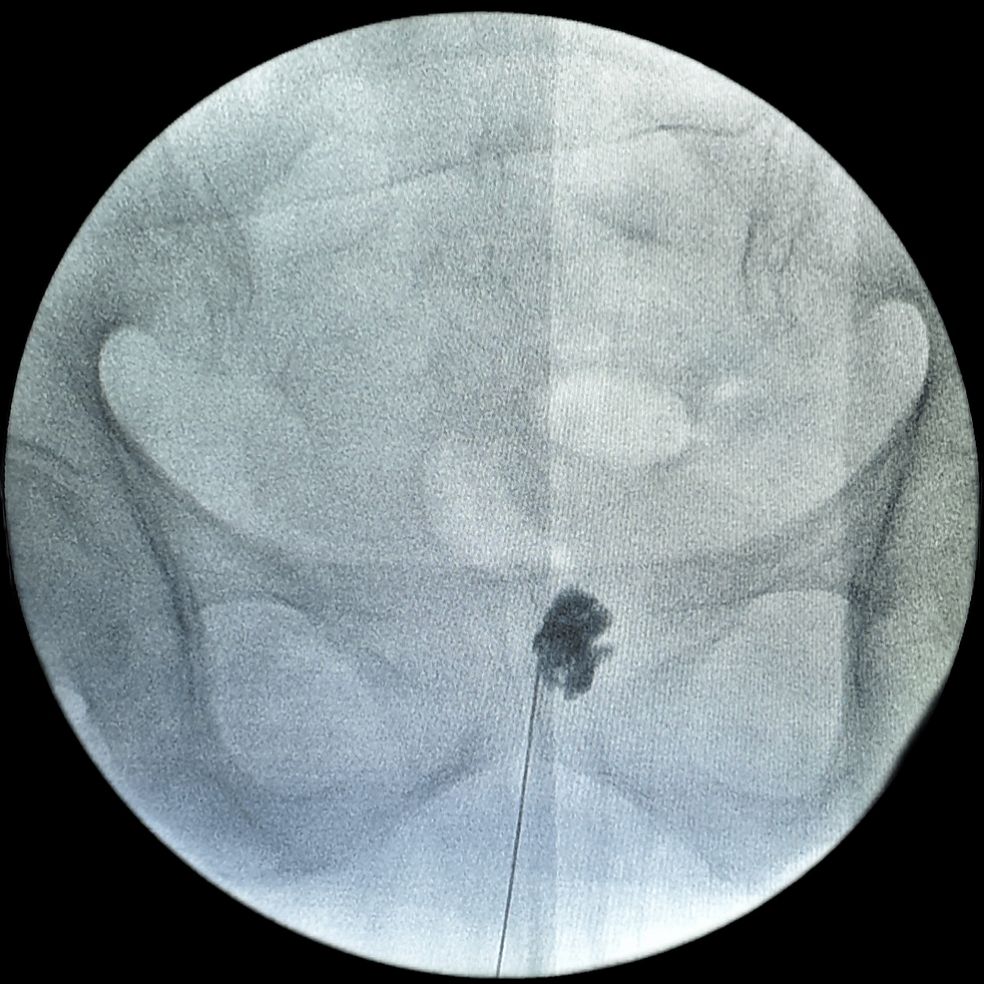
Anterior-posterior fluoroscopic image of the needle and injected contrast

**Figure 3:**
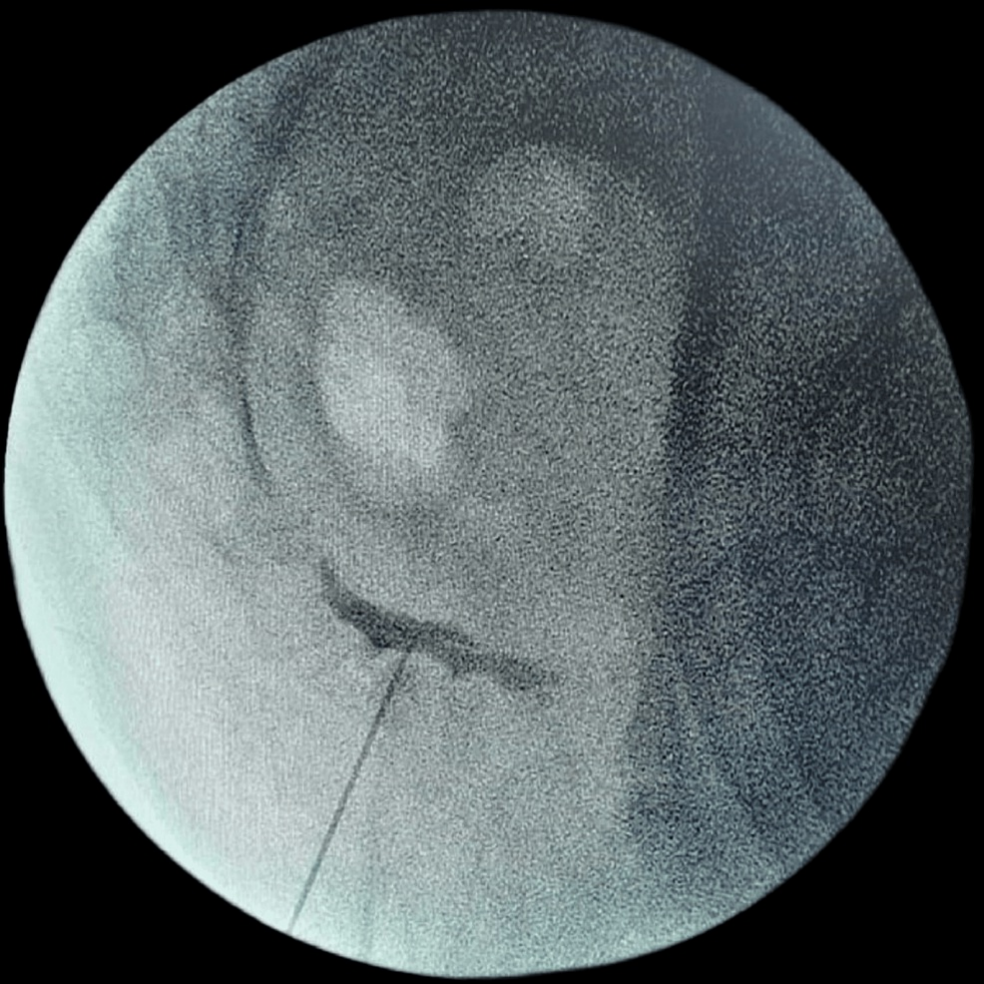
A lateral view of the fluoroscopic image showing the needle inserted at the intra-coccygeal joint and the injected contrast

The local coccygeal infiltration procedure was performed with the patient lying in a prone position with a pillow under the pelvis. The maximum tender point was palpated and marked. After sterile preparation of the area, skin infiltration was done with 2% lignocaine. Then a 22-gauge spinal needle was introduced under fluoroscopy, and 5 ml of Bupivacaine 0.25% with 40 mg of Methylprednisolone acetate was infiltrated around the soft tissue of the coccyx. In cases 1-3, ganglion impar block was given by the trans-sacrococcygeal technique. In the fourth case, the needle needed to pass through the intra-coccygeal joint because the sacrococcygeal disc was ossified and difficult to penetrate. The patients were positioned prone, as described in the local infiltration procedure. An anterior-posterior view of the coccyx was obtained by fluoroscopy to identify the midline. After sterile preparation and infiltration of the skin with Lignocaine 2%, a 22-gauge Quincke spinal needle was introduced until it hit the bone. The fluoroscope was now beamed into a lateral view to locate the sacrococcygeal and coccygeal joints. Then the needle was inserted either through the sacrococcygeal joint or the first intra-coccygeal joint until its tip reached the retroperitoneal space. The needle position was confirmed by injecting 0.2 ml of non-ionic contrast (Iohexol, Omnipaque). There was a linear contrast spread anterior to the coccyx and posterior to the rectum. It looked like an inverted comma and was described in the literature as a "comma sign" (Figures 1-3). After confirmation of needle position, 5 ml of 0.25% of bupivacaine with 40 mg of methylprednisolone acetate was injected, and the needle was withdrawn. Post-injection, firm pressure was applied over the injection site for hemostasis, and a sterile dressing was placed. The patients were advised to visit the pain clinic after a week for evaluation, and then follow-ups were done either physically or telephonically. No patients had any complications during or after the procedure.

## Discussion

Coccygodynia is frequently associated with subluxation and hypermobility of the sacrococcygeal joint [6]. Trauma to the coccyx due to falling backward is the commonest cause of coccygeal instability. The abnormal mobility of the coccyx tends to induce chronic inflammation, causing pain. Internal injury to the coccyx during a difficult instrumental delivery is also reported to cause coccygodynia [2]. Though coccygodynia is commonly associated with trauma, idiopathic coccygodynia is not uncommon. The coccyx is usually clinically normal in the latter. The inferred pathology may be due to degenerative joint disease, abnormal coccygeal anatomy, or repeated microtrauma to the coccyx due to prolonged sitting on hard surfaces [2]. The study by Adas et al. reported 29.3% of cases of idiopathic origin and 51.2% of coccygodynia with a history of trauma [10]. In another study by Galhom et al., trauma and idiopathic causes had equal incidences [7]. Women are more likely to develop coccygodynia due to the more posterior location of the sacrum and larger coccyx [4]. Obesity is another risk factor, and its association with coccygodynia is three times more common than in the general population [5]. Low back pain is commonly associated with coccygodynia, which can complicate the diagnosis and management. In the current series, one of the cases (the fourth case) presented with low back pain due to coccygodynia. Once the coccygodynia was taken care of, she was relieved of her low back pain.

There are no clear diagnostic criteria for coccygodynia. Clinical history and physical examination are sufficient to make a diagnosis. The perineum should be carefully inspected to rule out other etiologies of coccyx pain. Pilonidal cysts, pilonidal sinus, hemorrhoids, and a perineal abscess may also present with coccyx pain. The coccyx should be examined to look for sacrococcygeal joint instability. A rectal examination can be very informative as well. Elicitation of pain during mobilization of the coccyx rules in favor of nociceptive pain from referred pain originating from the lower pelvis. The Valsalva maneuver is typically suggested to establish the neuropathic component of coccygodynia. Aggravation of pain during the Valsalva maneuver indicates the neuropathic origin of coccygodynia [11]. Radiological investigations such as x-rays, CT scans, and MRIs are not routinely recommended for evaluating fractures unless there is a history of trauma. It may also reveal degenerative changes in the coccyx. Dynamic x-ray is particularly helpful in diagnosing sacrococcygeal joint instability [5].

There is a gamut of management strategies available to treat coccygodynia, which includes pharmacotherapy, physical therapy, minimally invasive injections, and surgical interventions. A trial of NSAIDs along with physical modalities such as sitz baths, the use of a coccygeal cushion (donut cushion), transcutaneous electrical nerve stimulation (TENS), sacrococcygeal joint mobilization, and levator ani relaxation exercises should be the first line of management [5]. Patients unresponsive to conservative therapy should be offered injection therapy. Soft tissue infiltration around the coccyx with local anesthetics with or without steroids is a simple intervention. It can be instituted early in combination with physical therapy to get maximum benefit [12]. As observed in our case series, it provided good short-term pain relief, but there were recurrences.

Ganglion impar block, on the other hand, can provide excellent, long-lasting pain relief, as demonstrated in the current case series and validated by previous studies [13-14]. The first description of ganglion impar block was given by Plancrate et al. for the treatment of perineal pain [15]. He used a bent needle to reach the retroperitoneal space behind the sacrococcygeal junction. However, Plancrate’s method did not become popular because his technique was technically challenging and marred by complications such as needle breaking, inadvertent rectal perforation, periosteal injection, and a high failure rate. To circumvent these technical challenges, Wenn and Serbeski introduced the trans-sacrococcygeal technique of the ganglion impar block, which is quicker and easier to perform [16]. In their study, they introduced the needle through the sacrococcygeal disc to reach the retroperitoneal space. There is a theoretical possibility of discitis, as there is a breach of the disc space in the trans-sacrococcygeal technique. Hence, strict asepsis must be maintained during the procedure. In the chemical neurolysis procedure, utmost care must be taken to prevent percutaneous spillage of the medication. Later, Munir et al. used a needle-inside-needle technique where they introduced a 22-gauge spinal needle into the sacrococcygeal disc. Through the 22-gauge needle, a 25-gauge needle is passed to reach the retroperitoneal space [17]. They argued that the needle-inside-needle technique significantly reduced rectal perforation, needle breakage, and discitis. In this case series, a single 22-gauge spinal needle was used and flushed with saline before being withdrawn after the procedure. The author advocates that in the case of chemical neurolysis procedures, the needle-inside technique may be beneficial in preventing the spillage of the neurolytic agent. Nevertheless, the needle should be flushed with saline before withdrawing from the sacrococcygeal joint.

Sometimes, in elderly patients, there is ossification of the sacrococcygeal disc, leading to difficulty in needle passage. In this situation, the needle can be passed through intra-coccygeal joints, as employed in the fourth case of the current series. The intra-coccygeal approach has been described previously as a proxy for the trans-sacrococcygeal technique [18]. Literature suggests that the ganglion impar is close to the first intra-coccygeal joint and can be blocked easily with less medication if the needle is introduced through the first intra-coccygeal joint. They further mentioned that the cornua of the first coccygeal bone often causes difficulty in the needle visualization in the lateral fluoroscopic view during the sacrococcygeal technique. In the intra-coccygeal approach, the needle tip can be visualized better as the cornua of the coccygeal bones are cephalad-angulated, and the rest of the other coccygeal bones do not have any cornu [18].

The paracoccygeal approach is another method that avoids the needle passing through the sacrococcygeal or intra-coccygeal joint [19]. It is a paramedian technique where ganglion impar is approached from the lateral side of the coccyx. In this technique, a bent spinal needle is used, which is manipulated by rotating clockwise and counterclockwise to reach the retroperitoneal space. In our opinion, the intra-coccygeal approach is challenging because the joints are too small. It should only be tried when the trans-sacrococcygeal joint approach is not feasible. The paracoccygeal technique can also be considered, but it requires multiple needle manipulations that may be uncomfortable for the patient.

Recurrence of pain after a single-shot ganglion impar block in coccygodynia is not uncommon. It can be managed by either a repeat block or radiofrequency ablation treatment. The latter can be easily applied to get long-term pain relief [10]. Chemical neurolysis of ganglion impar using phenol or alcohol is usually reserved for managing intractable malignant pain [9]. Coccygectomy is the surgical treatment of coccygodynia. The common indications for surgical interventions are coccygeal instability, subluxation, and coccygeal spicule [6]. In the current series, a single injection of ganglion impar block was enough to provide long-term pain relief in all patients. If coccygodynia is not treated early, it may lead to the onset of chronic pain with profound emotional and psychological effects. So, patients with long-standing coccygodynia should be approached holistically with combinations of injection therapy, physical therapy, and psychosocial treatment.

## Conclusions

In most patients, idiopathic coccygodynia is usually self-remitting and responds to physical therapy, ergonomic modifications, and medications. Some of them may develop chronic pain and respond poorly to conservative management. In these patients, a combination of minimally invasive injections and physical therapy can significantly reduce pain and improve quality of life. Ganglion impar block is one of the minimally invasive injection modalities that can be employed for managing coccygodynia. The procedure can be easily performed under fluoroscopy guidance, and a single-shot ganglion impar block can provide excellent long-term pain relief. The trans-sacrococcygeal technique of ganglion impar block is usually the technique of choice, where the needle must pass through the sacrococcygeal disc. Intra-coccygeal and paracoccygeal techniques are a few other fluoroscopic methods described in the literature. These alternative methods can come into play in challenging scenarios, especially in elderly patients with an ossified sacrococcygeal joint.
